# Collagen type I promotes osteogenic differentiation of amniotic membrane-derived mesenchymal stromal cells in basal and induction media

**DOI:** 10.1042/BSR20201325

**Published:** 2020-12-14

**Authors:** Haselamirrah Mohd Akhir, Peik Lin Teoh

**Affiliations:** Biotechnology Research Institute, Universiti Malaysia Sabah, Jalan UMS, Kota Kinabalu 88400, Sabah, Malaysia

**Keywords:** adipogenesis, Collagen, mesenchymal stromal cell, osteogenesis, stemness, surface markers

## Abstract

Collagen has been widely shown to promote osteogenesis of bone marrow mesenchymal stromal cells (BM-MSCs). Due to the invasive procedure of obtaining BM-MSCs, MSCs from other tissues have emerged as a promising alternative for regenerative therapy. MSCs originated from different sources, exhibiting different differentiation potentials. Therefore, the applicability of collagen type I (COL), combining with amniotic membrane (AM)-MSCs was examined through proliferation and differentiation assays together with the expression of surface markers and genes associated with stemness and differentiation under basal or induction conditions. No increase in cell growth was observed because AM-MSCs might be directed toward spontaneous osteogenesis. This was evidenced by the calcium deposition and elevated expression of osteogenic genes when AM-MSCs were cultured in collagen plate with basal media. Under the osteogenic condition, reciprocal expression of *OCN* and *CEBPA* suggested a shift toward adipogenesis. Surprisingly, adipogenic genes were not elevated upon adipogenic induction, although oil droplets deposition was observed. In conclusion, our findings demonstrated that collagen causes spontaneous osteogenesis in AM-MSCs. However, the presence of exogenous inductors could shift the direction of adipo-osteogenic gene regulatory network modulated by collagen.

## Introduction

Mesenchymal stromal cells (MSCs) have been traditionally isolated from the bone marrow. However, due to its invasive and painful procedure for the donor, MSCs derived from adult tissues such as adipose, amniotic membrane (AM), umbilical cord (UC) have been used as alternative sources for tissue engineering and regenerative medicine. Besides that, they have less ethical constraints, more accessible and low immunogenicity [1]. In addition to the prominent roles of MSCs for regenerative medicine, their profound functions as medicinal signalling cells have recently attracted great interest.

Nonetheless, MSCs need to be manipulated *in vitro* to obtain enough cell number for therapeutic purposes. To achieve this, natural or synthetic biomaterials have been extensively investigated for their potentials in maintaining stemness and directing the differentiation of MSCs [2–4]. Collagen type 1, a protein abundantly found in the extracellular matrix has been broadly shown to promote proliferation, survival, adhesion and osteogenesis in bone marrow MSCs [5,6]. It has also been demonstrated to promote endoderm differentiation of human embryonic stem cells [7]. However, the effects of collagen on other MSCs such as AM-MSCs are not well studied.

AM-MSCs as a promising source have been shown to be capable of self-renewal and multilineage differentiation. Emerging evidence has revealed that MSCs from different origins or culture conditions exhibiting discrepancy in term of their biological potentials such as proliferation, differentiation capability as well as gene expression profile [8–10]. It has been reported that AM-MSCs and UC-MSCs exhibited differences in cell growth and gene expression patterns [11]. For instance, AM-MSCs were shown to have the highest osteogenic potential compared with other neonatal MSCs [10]. Conversely, Araújo et al. demonstrated that neonatal MSCs were more efficient toward osteogenesis than adipogenesis [12]. Therefore, there is a necessity to evaluate how biomaterials modulate different types of MSCs before utilizing them for therapeutic purposes. In the present study, we examined the modulating effects of collagen type I (COL) on AM-MSCs covering the aspects of morphology, proliferation, differentiation, as well as the expression profiles of genes governing immunophenotyping, stemness and adipo-osteogenic commitment.

## Materials and methods

### Sample collection

The study was approved by the Ethics and Research Committee of Universiti Malaysia Sabah with approval code: JKEtika 1/16 (1). The informed consent was also obtained from patients. Full-term placenta from healthy newborn delivered by cesarean section was collected from Damai Specialist Hospital and KPJ Sabah Specialist Hospital, Kota Kinabalu. The samples were transferred immediately to the laboratory for cell isolation.

### Isolation of AM-MSCs

The isolation process of AM-MSCs was done according to the isolation method published by Fatimah et al. [13] with some modifications. AM was peeled off aseptically from the placenta and washed with 1× Dulbecco’s phosphate-buffered saline (DPBS) (Gibco-Invitrogen, Carlsbad, CA, U.S.A.) several times to remove red blood cells. Then the membrane was cut into small pieces with a size of 2 × 2 cm^2^. The tissues (20 pieces) were proceeded to serial digestions with 0.05% trypsin-EDTA (Gibco-Invitrogen, Carlsbad, CA, U.S.A.) for 10 min initially followed by another three-times digestion for 30 min each. After that, the denuded membranes were washed with 1× DPBS followed by digestion using 0.1% collagenase type I (Worthington, Minnesota, U.S.A.) for 40 min at 37°C with constant agitation at 180 rpm. The cell suspension was collected and centrifuged at 5000 rpm for 5 min at room temperature. The resulting cell pellet was resuspended with culture medium and transferred to a 25-cm^2^ tissue culture flask.

### Cell culture, cell count and morphological observation

Isolated cells were cultured using Dulbecco’s Modified Eagle’s Medium/Nutrient Mixture F-12 (DMEM/F12) (Gibco-Invitrogen, Carlsbad, CA, U.S.A.) containing 10% fetal bovine serum (Gibco-Invitrogen, Carlsbad, CA, U.S.A.), 1% GlutaMAX (Gibco-Invitrogen, Carlsbad, CA, U.S.A.), 1% ascorbic acid (Merck, Darmstadt, Germany) and 1% antibiotic–antimycotic (Gibco-Invitrogen, Carlsbad, CA, U.S.A.). Medium was changed every 3 days until cells reached 80–90% confluency. This was followed by subculturing using 0.125% trypsin-EDTA. Cells were also cultured on microplates coated with COL (#152034: 6-well plates, #152036: 96-well plates) purchased from Thermo Fisher Scientific (Waltham, MA, U.S.A.). Cell viability assay was determined using 0.4% Trypan Blue solution (Gibco-Invitrogen, Carlsbad, CA, U.S.A.). Cells at specific passages were trypsinized at day 7 to obtain total cell number and their morphology at early, and late passage (P) was observed using an inverted microscope.

### Cell proliferation assay

Cell proliferation assay was performed using PrestoBlue Cell Viability Reagent (Thermo Fisher Scientific, Waltham, MA, U.S.A.) according to the manufacturer’s procedure. Cells at a density of 5000 cells/well were seeded in 96-well microplates coated with or without collagen. After overnight incubation, the PrestoBlue solution was added to the cells and incubated for 1 h at 37°C in the dark. The fluorescence signal was measured at the excitation wavelength of 560 nm and an emission wavelength of 590 nm. The measurement was obtained every 2 days. Data were obtained from at least three independent experiments with each of them being performed in triplicates.

### Differentiation assays

A total of 5.0 × 10^3^ cells were first grown on 96-well plate, then induced at day 3 using StemPro osteogenesis and StemPro adipogenesis differentiation kit (Gibco-Invitrogen, Carlsbad, CA, U.S.A.). Media were changed every 3 days, and the cells were cultured for 2–3 weeks. After induction, cells were washed with 1× DPBS and fixed with 10% formalin (Merck, Darmstadt, Germany) for 20 min at room temperature. Calcium deposits and lipid droplets were stained using Alizarin Red S (ScienCell Research Laboratories, Carlsbad, CA, U.S.A.) and Oil Red O (Merck, Darmstadt, Germany), respectively, according to the manufacturers protocols. After washing, cells were observed under an inverted microscope.

### RNA extraction and RT-PCR

Cells with a density of 3 × 10^4^ cells were grown in each well of six-well microplate. Total RNA was extracted using TransZol Up Plus RNA Kit (Transgen Biotech, Beijing, China). RT-PCR and RT-qPCR were carried out using TransScript One-Step RT-PCR SuperMix (Transgen Biotech, Beijing, China) and OneStep iTaq Universal SYBR Green (Bio-Rad Laboratories, Irvine, CA, U.S.A.), respectively. Procedures were performed following the manufacturer’s instructions, except the annealing step that was performed at 55–58°C. Primers targeting stemness and differentiation genes were listed in Table 1. Surface markers were synthesized based on sequences published by Ali et al. [14]. PCR products were observed through 2% agarose gel electrophoresis. For semi-quantitative PCR, the gene expression levels in each sample were normalized to their respective *β-actin* expression levels and compared with control. For RT-qPCR, the relative expression was obtained by calculating fold change using the 2^−ΔΔ*C*_t_^ method for each pairwise analysis. Fold change ≥ 1.5 is considered a significant increase or decrease in expression.

**Table 1:** List of sequences of forward (F) and reverse (R) primers used in PCR

Genes	Primer sequences
*OCT4*	F 5′-TGAGTAGTCCCTTCGCAAGC-3′
	R 5′-TTAGCCAGGTCCGAGGATCA-3′
*NANOG*	F 5′-ACCAGAACTGTGTTCTCTTCCACC-3′
	R 5′-CCATTGCTATTCTTCGGCCAGTTG-3′
*RUNX2*	F 5′-GACCAGTCTTACCCCTCCTACC-3′
	R 5′-CTGCCTGGCTCTTCTTACTGAG-3′
*OCN*	F 5′-GTGCAGAGTCCAGCAAAGGT-3′
	R 5′-TCAGCCAACTCGTCACAGTC-3′
*C/EBPB*	F 5′-TTTGTCCAAACCAACCGCAC-3′
	R 5′-GCATCAACTTCGAAACCGGC-3′
*C/EBPA*	F 5′-GCAAACTCACCGCTCCAATG-3′
	R 5′-CTTCTCTCATGGGGGTCTGC-3′
*ACTB*	F 5′- GTCATTCCAAATATGAGATGCGT-3′
	R 5′- GCTATCACCTCCCCTGTGTG-3′

### Statistical analysis

All experiments were performed using three independent biological samples and expressed as mean ± standard deviation (SD). Statistical analysis was done using paired *t* test, and *P*-value <0.05 was considered significant.

## Results

### Morphology and proliferation of AM-MSCs

**Figure 1:**
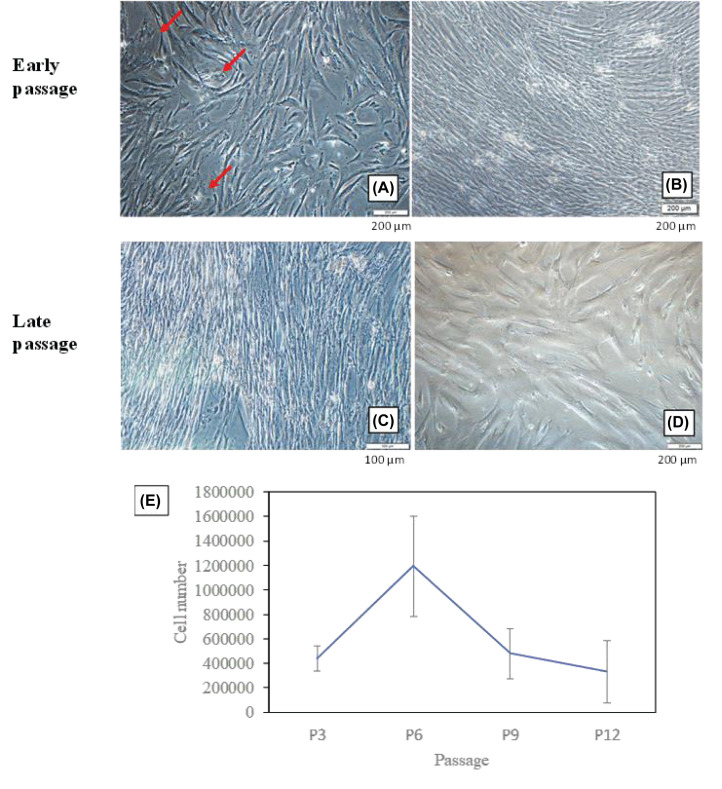
The morphology and proliferation of AM-MSCs at early and late passages (**A**) P3, (**B**) P6, (**C**) P9 and (**D**) P12. (**E**) Cell numbers for each passage were calculated from three independent samples with triplicates. Red arrows indicate round-shaped cells.

Due to the heterogeneity of freshly isolated MSCs, we observed cell morphology from early to late passages. At P3 (Figure 1A), spindle-shaped cells and a few round-shaped cells (red arrows) were seen in the culture. However, starting from P6 onward, we noticed the disappearance of round-shaped cells (Figure 1B–D). From our observation, if the majority of the isolated cells were appeared in round-shaped, the chances for cells to survive for longer passages was low (data not shown). Besides, cells at P12 became larger and longer in shape (Figure 1D). From the proliferation assay (Figure 1E), cells at P6 had the highest proliferation compared with other passages. A sharp decrease in cell count was found at late passages (P9 and P12). As the success rate of isolated cells proliferated beyond P10 was low, we had chosen P6 and P9 to represent early and late passages for further analysis.

### Surface marker expression and differentiation potentials of AM-MSCs

**Figure 2:**
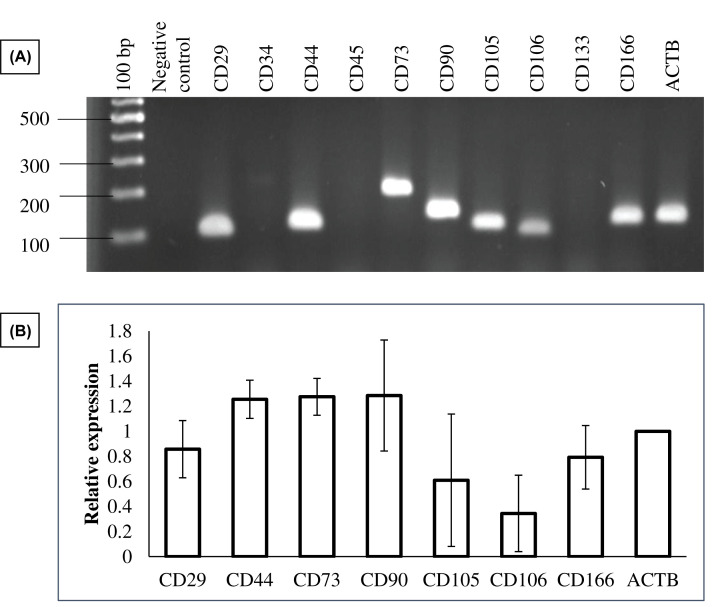
The mRNA expression of surface markers in AM-MSCs (**A**) A representative agarose gel photo obtained by RT-PCR. (**B**) Relative expression of positive CD markers.

**Figure 3:**
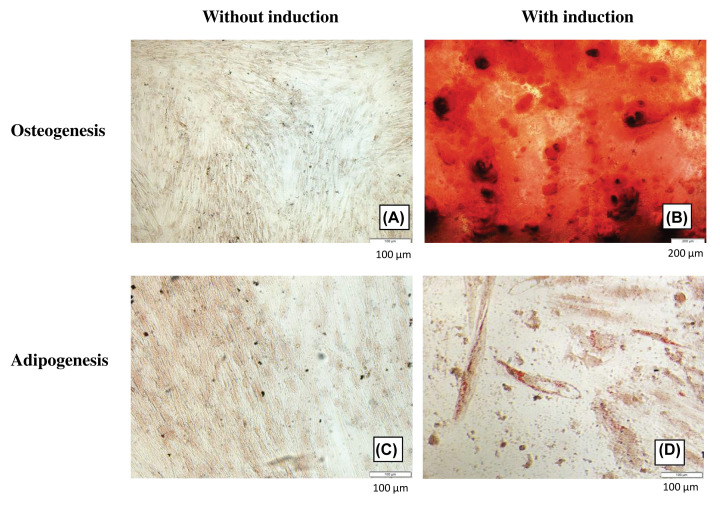
Differentiation potentials of AM-MSCs Cells cultured in the basal and induction media were stained Alizarin Red S (**A**,**B**) and Oil Red O (**C**,**D**), respectively.

To confirm the identity of isolated cells, differentiation potentials and the mRNA expression of surface markers specific to MSCs were assessed (Figure 2). As expected, these cells displayed negative expression of both hematopoietic markers (*CD34, CD45*) and endothelial markers (*CD133*). Consistent with previous studies [10,15], isolated cells expressed all positive MSC markers: *CD29, CD44, CD73, CD90, CD105*, and *CD166* which are frequently used for confirming the status of MSCs. Although the expression of *CD106* is controversial, low expression of this surface marker has been reported in AM-MSCs [16]. CD271 which expresses in AM-MSCs but not epithelial stem cells [17] was confirmed through immunofluorescence staining (unpublished data). The presence of calcium deposition in osteocytes (Figure 3B) and lipid accumulation in adipocytes (Figure 3D) suggested that the isolated cells could differentiate into osteogenic and adipogenic lineages upon induction compared to the controls (Figure 3A,B).

### Effects of collagen on cell morphology, proliferation and surface markers

**Figure 4:**
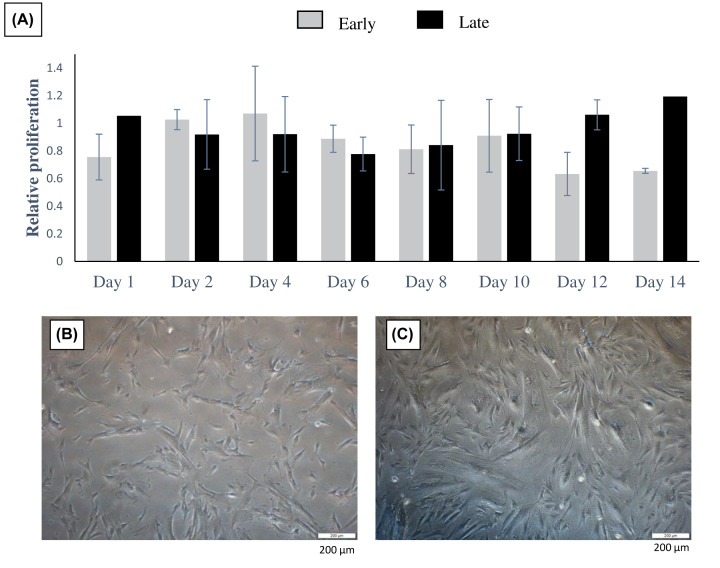
The effects of collagen on cell proliferation and morphology (**A**) Relative cell proliferation of AM-MSCs cultured on collagen to control. The morphology of cells cultured on collagen at early (**B**) and late (**C**) passage.

**Figure 5:**
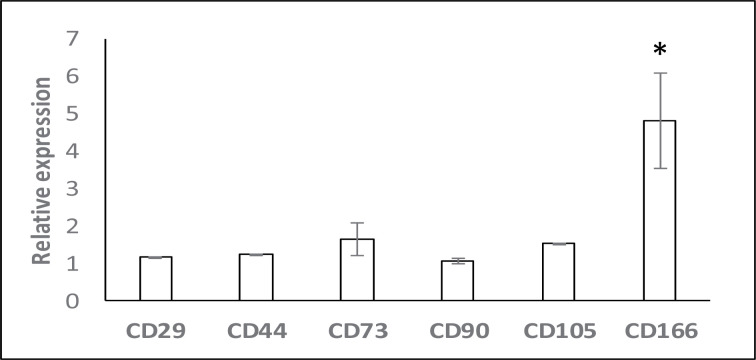
The effects of collagen on positive surface markers Semi-quantitative analysis of CD markers expression. Asterisk (*) indicates statistical significance.

To ascertain the possible effect of collagen (COL) on the proliferation of AM-MSCs, cells were cultured on the collagen-coated plate (AM-MSCs-COL). As shown in Figure 4A, AM-MSCs-COL at the early passage showed comparable cell growth with the control as no obvious changes were observed up to day 10. However, drastic decreases in cell proliferation were shown on days 12–14. On the other hand, the growth pattern at late passage was steadily maintained throughout the culturing periods. We also found that some samples could survive up to 20 days while cells at the early passage were mostly dead and detached (data not shown). This indicated that collagen potentially prolongs cell survival, especially at the later passage, but more sample number is required to confirm this. No apparent difference between the early and late passages in term of cell morphology except cells looked less dense compared with those in collagen plate after 10 days (Figure 4). Among the positive surface markers, only the expression of *CD166* was significantly increased to five-fold when compared with control (Figure 5). The gene expression of other surface markers was mostly unaltered in the presence of collagen.

### Collagen promotes osteogenic differentiation

**Figure 6:**
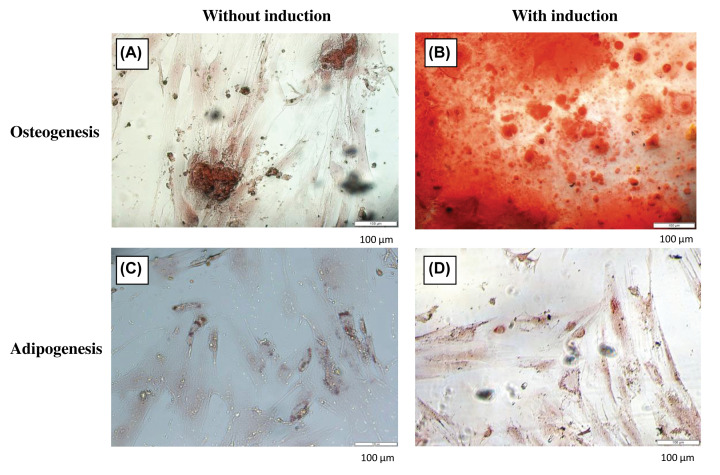
The effects of collagen on osteogenic and adipogenic differentiation of AM-MSC Cells cultured in the basal and induction media were stained with Alizarin Red S (**A**,**B**) and Oil Red O (**C**,**D**), respectively.

We further investigated whether collagen influences lineage commitment of AM-MSCs in the presence of exogenous inductors or without. From our results, we noticed that collagen was capable of inducing osteogenesis spontaneously in basal media (Figure 6A,C). AM-MSCs retained their osteogenic and adipogenic differentiation potentials when cultured in collagen plates (Figure 6B,D). The increase in calcium deposition was observed in AM-MSCs-COL (Figure 6B). Besides that, collagen had also caused more oil droplets accumulation upon adipogenic induction (Figure 6D). These results indicated a potential synergistic effect of collagen and exogenous inductors.

### Effects of collagen on the expression of stemness and differentiation genes

**Figure 7:**
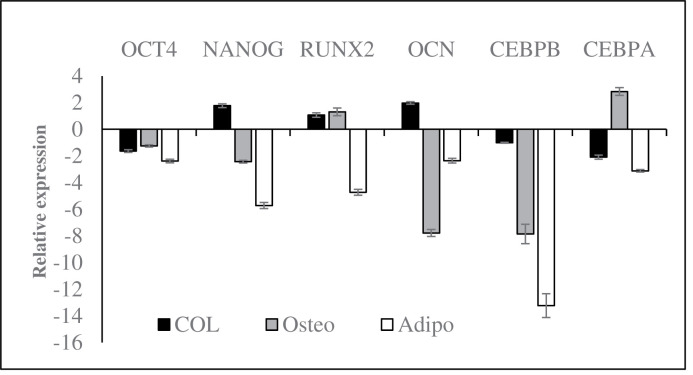
Collagen altered the expression of genes associated with stemness and differentiation in basal and induction media

To confirm the observation shown in differentiation caused by collagen, we determined the expression levels of genes associated with stemness and differentiation. In basal media (Figure 7, dark), collagen was found to suppress the expression of *OCT4* while increasing the expression of osteogenic genes (*RUNX2* and *OCN*). This was in line with the spontaneous osteogenesis we observed above. We noticed the down-regulation of *OCT4, NANOG, OCN* and *CEBPB* in AM-MSCs-COL, while the expression of *RUNX2* and *CEBPA* was up-regulated when cells were cultured in osteogenic media (Figure 7, grey). Surprisingly, the expression of *CEBPA* and *CEBPB* was down-regulated even in the presence of adipogenic media (Figure 7, white). This finding was negatively correlated with the oil deposition observed in adipogenesis assay. Nevertheless, this is insufficient to conclude that the inhibition of adipogenesis in AM-MSCs-COL as the gene regulatory may be activated via unknown mechanisms.

## Discussion

Biomaterials with the ability to enhance proliferation and direct desired lineage differentiation are promising in overcoming inherent bottlenecks of MSCs. Comparative analysis of MSCs obtained from various sources has uncovered their distinct physiological properties that might restrict or favor their functional roles in a specific application. Therefore, combining biomaterials with different MSCs may have divergent implications on gene regulatory network. AM is an appealing source of MSCs owing to its high accessibility and high recovery rate and multilineage potentials [18,19]. Collagen has been widely used as a scaffold and shown in directing osteogenic commitment in bone marrow mesenchymal stromal cells (BM-MSCs). In the present study, freshly isolated AM-MSCs at early and late passages were subjected to the examination of cell morphology, expression of MSC surface markers and differentiation potentials to confirm their stemness properties. Based on the morphological and proliferation studies, AM-MSCs at early passage was used for further investigation to avoid inconsistent results

After ensuring the identity of isolated cells, they were applied on the collagen surface. Our results showed not many differences in term of proliferation when compared with cells cultured on tissue culture plate (control). However, this matrix has been shown to promote the proliferation of other MSCs [6,20]. From Alizarin staining, we noticed calcium deposition in AM-MSCs-COL without exogenous induction, suggesting the occurrence of spontaneous differentiation. Both collagen alone or collagen with other biomaterials have also been shown to induce osteogenesis in the 2D culture of human MSCs [21–24]. Besides, spontaneous differentiation was also observed in 3D microcarriers [24]. Similarly, this is in line with previous studies showing the reciprocal relationship between proliferation and differentiation in the basal medium [5,25].

On another note, our results showed that collagen elevated *CD166* expression in AM-MSCs, but there was no significant alteration in the expression of other positive markers. CD166 (activated leucocyte cell adhesion molecule, ALCAM) is a transmembrane glycoprotein that belongs to the immunoglobulin superfamily [26]. They are cell adhesion molecules, thus increase in its expression could be due to AM-MSCs attached more tightly to collagen matrix as demonstrated by more closely packed morphology in Figure 5. CD166 has also been identified as cancer or cancer stem cell marker and involved in metastasis of various tumors [27–29]. However, only a few studies indicated the link between CD166 and other surface markers. For instance, reduction in CD166 and CD44 expression was observed in shRNA CD90 MSCs, leading to osteogenic differentiation in amniotic fluid, dental pulp and adipose MSCs [30]. However, collagen did not alter the expression levels of *CD44* and *CD90* in our study and also MSC markers mentioned by Parolini et al. [31]. High expression of CD166 surface marker was only reported in osteoarthritis (OA) MSCs showing increased expression of cartilage markers *RUNX2* and *COL10A1*. It was hypothesized that OA-MSCs drive the OA progression [32]. To date, the role of CD166 in MSCs is still unclear.

Furthermore, the occurrence of spontaneous osteogenesis was supported by the up-regulation of osteogenic genes (*RUNX2* and *OCN*). In contrast, the adipogenic genes (*CEBPB* and *CEBPA*) and *OCT4* were simultaneously down-regulated. Although NANOG is also one of the master regulators of pluripotency, suppression of *NANOG* expression was not observed in AM-MSCs-COL cultured in basal media. It is well known that OCT4, SOX2 and NANOG are prominent in maintaining pluripotency of ESCs and MSCs despite conflicting findings [33–35]. According to Pierantozzi et al., *NANOG* was only detected in MSCs after *in vitro* expansion, and its expression was not associated with proliferative or differentiation potentials of MSCs [34]. The overexpression of NANOG was found to promote the proliferation and osteogenesis of human MSCs but impeded its commitment toward adipogenesis [36]. In contrast, the decrease in proliferation and differentiation of human dental pulp stem cells were observed after depletion of OCT4 and NANOG [37]. These findings demonstrated that NANOG might play a role in regulating lineage differentiation.

When examining the implication of osteogenic media on AM-MSCs-COL, we noticed the decreased *NANOG* and *OCN* expression levels were accompanied by an increase in the expression of *CEBPA*. This demonstrates a potential shift from differentiated osteocytes to adipocyte [38], as less calcium deposition was seen in Alizarin Red stained AM-MSCs. A parallel event of adipo-osteogenesis upon BMP2 induction has been reported by Ponce et al. [39], suggesting inverse expression of osteogenic and adipogenic genes was not exclusive.

Surprisingly, the down-regulation of adipogenic genes (*CEBPA* and *CEBPB*) was observed when AM-MSCs-COL was induced with adipogenic media despite seeing oil droplets in Oil Red stained cells (Figure 7D). Dexamethasone (Dex) has been shown to inhibit osteogenesis by suppressing the expression of *OCN* through PI3K/Akt signalling pathway [40–42]. Taken together, we suspect that the presence of exogenous factors such as Dex in induction media triggers a directional switch in differentiated AM-MSCs-COL, causing imbalance and dysregulation of adipo-osteogenic gene regulatory. The roles of Dex in trans-differentiation of MSCs have been shown in many studies [43,44]. It has been reported that Dex shifted osteoblastogenesis of BM-MSCs to adipogenesis by up-regulating the expression of *CEBPA* through DNA hypomethylation [45]. Besides that, collagen is known as the extracellular matrix protein, providing RGD binding sites for different types of integrin receptor depending on cell type [46]. Besides, a synergistic effect between collagen and inductors could not be excluded. Thus, this may also create an extracellular landscape different from the typical scenario leading to distinct gene regulatory network. Nonetheless, the possibility of collagen in directing lineage commitments through its topography should not be neglected as revealed by many studies [47,48].

## Conclusion

We have demonstrated that collagen is capable of inducing spontaneous osteogenesis of AM-MSCs in exogenous inductors free condition without affecting their intrinsic properties in adipo-osteogenic differentiation by modulating associated gene pathways. This makes it a promising biomaterial for clinical application.

## Data Availability

The data that support the findings of the present study are available upon request.

## Abbreviations

AM: amniotic membrane
BM-MSC: bone marrow mesenchymal stromal cell
BMP2: bone morphogenetic protein 2
COL: collagen type I
Dex: Dexamethasone
DPBS: Dulbecco’s phosphate-buffered saline
MSC: mesenchymal stromal cell
OA: osteoarthritis
RT-qPCR: quantitative reverse transcription polymerase chain reaction
UC: umbilical cord
